# Perspectives of pregnant and breastfeeding women on longitudinal clinical studies that require non-invasive biospecimen collection – a qualitative study

**DOI:** 10.1186/s12884-021-03541-x

**Published:** 2021-01-20

**Authors:** Dominick J. Lemas, Lauren Wright, Elizabeth Flood-Grady, Magda Francois, Lynn Chen, Austen Hentschel, Xinsong Du, Chu J. Hsiao, Huan Chen, Josef Neu, Ryan P. Theis, Elizabeth Shenkman, Janice Krieger

**Affiliations:** 1 Department of Health Outcomes and Biomedical Informatics, College of Medicine, University of Florida, Gainesville, USA; 2 Department of Obstetrics & Gynecology, College of Medicine, University of Florida, Gainesville, USA; 3 Clinical Translational Science Institute, University of Florida, Gainesville, USA; 4 STEM Translational Communication Center, College of Journalism and Communications, University of Florida, Gainesville, USA; 5 MD-PhD Training Program University of Florida, Gainesville, USA; 6 Genetics Institute, University of Florida, Gainesville, USA; 7 Department of Anthropology, University of Florida, Gainesville, USA; 8 Department of Advertising, College of Journalism and Communications, University of Florida, Gainesville, USA; 9 Department of Pediatrics, College of Medicine, University of Florida, Gainesville, USA

**Keywords:** Qualitative research, Thematic analysis, Grounded theory, Mother-infant, NVivo, Study visits, Stool samples

## Abstract

**Background:**

Investigation of the microbiome during early life has stimulated an increasing number of cohort studies in pregnant and breastfeeding women that require non-invasive biospecimen collection. The objective of this study was to explore pregnant and breastfeeding women’s perspectives on longitudinal clinical studies that require non-invasive biospecimen collection and how they relate to study logistics and research participation.

**Methods:**

We completed in-depth semi-structured interviews with 40 women who were either pregnant (*n* = 20) or breastfeeding (*n* = 20) to identify their understanding of longitudinal clinical research, the motivations and barriers to their participation in such research, and their preferences for providing non-invasive biospecimen samples.

**Results:**

Perspectives on research participation were focused on breastfeeding and perinatal education. Participants cited direct benefits of research participation that included flexible childcare, lactation support, and incentives and compensation. Healthcare providers, physician offices, and social media were cited as credible sources and channels for recruitment. Participants viewed lengthy study visits and child protection as the primary barriers to research participation. The barriers to biospecimen collection were centered on stool sampling, inadequate instructions, and drop-off convenience.

**Conclusion:**

Women in this study were interested in participating in clinical studies that require non-invasive biospecimen collection, and motivations to participate center on breastfeeding and the potential to make a scientific contribution that helps others. Effectively recruiting pregnant or breastfeeding participants for longitudinal microbiome studies requires protocols that account for participant interests and consideration for their time.

**Supplementary Information:**

The online version contains supplementary material available at 10.1186/s12884-021-03541-x.

## Background

In the past, pregnant or breastfeeding mothers were excluded from participating in clinical research due to potential adverse outcomes [1]. An increasing number of clinical investigations involving this population, however, has been stimulated by the emerging importance of the microbiome. For example, exclusive breastfeeding through the first 6 months of life is associated with positive maternal-infant health outcomes [2], and breastfeeding modifies the function of the infant microbiome and may influence infant health outcomes [3]. The microbiome, which consists of the entire habitat of gut microbiota including microorganisms and microbial products [4], plays a critical role in human health and disease [5]. Postnatal development of the infant microbiome occurs primarily through maternal-infant interaction [6]; this interaction can be impacted by factors including mode of delivery [7], maternal obesity [8], antibiotics [9], and infant diet [10].

The increasing clinical investigations with pregnant or breastfeeding women involve collection of biospecimens such as stool and human milk [11, 12]. Stool samples are used to evaluate the gut microbiome [13] and human milk samples to identify novel compounds that interact with the infant gut microbiome [14]. Despite the growing appreciation for the gut microbiome as a factor that mediates the health benefits of breastfeeding [14], recruitment of participants for clinical microbiome studies that require biospecimen collection can be difficult, particularly when there is no obvious benefit to the participant [15].

Relatively little is known about recruitment and retention of pregnant and breastfeeding women into clinical microbiome studies that involve biospecimen collection. One study on stool sampling in elderly men indicated practical methods for collecting biospecimens are warranted for successful recruitment [4]. To address the gap in knowledge about recruitment of pregnant and breastfeeding women in clinical studies that require biospecimen collection, we conducted a qualitative study to explore their understanding of longitudinal clinical research, motivations and barriers to their participation in studies, and their preferences related to non-invasive biospecimen sampling.

## Methods

Snowball sampling was used to recruit participants (*N *= 40) from Gainesville, Florida. Channels such as word-of-mouth, flyers, and social media were used to initiate an inclusive community-based means of participant recruitment. Participants were female, between 18 and 45 years of age, and either currently pregnant (*n* = 20) or actively breastfeeding an infant younger than 12 months of age (*n* = 20). Exclusion criteria and recruitment methods for participation mirrored a larger ongoing longitudinal microbiome study. Participants were excluded if they had only one breast capable of producing milk or a history of any of the following: inadequate milk production, pre-eclampsia, pre-term delivery, or habitually consuming alcohol, drugs, or tobacco during pregnancy.

Prior to conducting semi-structured interviews with participants, they all received a biospecimen collection kit with instructions to collect saliva, urine, a vaginal swab stool, and human milk from mothers who were breastfeeding. Self-collection of samples was optional. An interview guide was used to facilitate discussion during the interviews [16]. Questions focused on preferences for and on facilitators and barriers to participating in research studies. Interviews were conducted face-to-face in a private room and lasted between 30 and 60 min. All interviews were audio recorded with participant consent and were later professionally transcribed (Datagain, Secaucus, NJ). Supplementary Fig. 1 outlines the recruitment strategies and enrollment outcomes for this study.

We used grounded theory as a model for our analysis [17]. Briefly, during the first stage of data analysis, three coders read through a portion of the transcripts (10%) and assigned a priori codes reflecting the major categories drawn from our interview guide: participant background, participating in research studies, and biological sample collection. A second stage of analysis was performed to establish coder inter-reliability during which coders were assigned four of the same transcripts to code independently. Next, theme level of agreement among coders was evaluated using NVivo coding stripes [18]. Discrepancies in coding were discussed among the coders until consensus was achieved. Some sub-themes for facilitators and barriers associated with research participation were adapted from previous work in this population [15], whereas other new sub-themes emerged from data.

## Results

Study recruitment included 103 encounters with potential participants (e.g., by phone or email) and 77 phone screenings, of which 63 individuals passed. We consented 47 participants, 40 of which completed the semi-structured interview.

### Participant characteristics

Table 1 presents the demographics of participants who completed the semi-structured interviews (*N* = 40). On average, participants were between 31 and 40 years of age (*n* = 24, 60%), and a majority had a professional and/or graduate degree. Participants included 22 second time mothers and most reported previous experience with biomedical or social science research. Only 1 in 5 participants reported previous experience using a stool collection kit. Just over half of the participants donated all biological samples requested (*n* = 23, 57.5%), and 29 (72.5%) participants donated at least one biological sample.

**Table 1:** Participant Demographic Information^a^

Characteristic	Frequency (n)	Percent (%)
**Maternal age**		
20–30 years	16	40
31–40 years	24	60
**Previous children**		
Yes	22	55
No	15	37.5
**Highest level of education**		
Professional/graduate degree	21	52.5
Some graduate education	3	7.5
College degree	9	22.5
Some college education	1	2.5
Associate degree	3	7.5
Technical/vocational degree	2	5
**House-hold income**		
0 to $37,000	5	12.5
$37,001 to $79,000	14	35
$79,001 or more	16	40
**Pre-pregnant body mass index (kg/m2)**		
< 25	26	65
25–30	8	20
➢ 30	6	15
**Previous research experience**		
Yes	22	55
No	17	42.5
**Previous experience with stool collection**		
Yes	9	22.5
No	21	52.5

^a^7.5% of data were missing for the previous children and highest level of education variables, 12.5% of data were missing for the house-hold income variable, 2.5% of data were missing from the previous research experience variable, and 25% of data were missing from previous experience with stool collection variable

### Study preferences

Table 2 summarizes participant time and communication preferences for participating in longitudinal clinical microbiome studies. Out of 40 participants, greater than half said they would generally be unconcerned with the duration of the study and would participate in studies longer than 24 months. Participants would be comfortable participating in studies that required more than four study visits but were adamant that each visit should last no more than 60 min. Participants would prefer text messaging for communication with the study team, would generally be interested in scheduling study visits in the morning, and would prefer study reminders be sent either 1–2 days in advance or 6–10 days before a study visit.

**Table 2:** Participant Study Preferences^a, b^

Study Preferences	Frequency (n)	Percent (%)
**Study duration (months)**		
0–6	2	5
7–12	4	10
13–24	3	7.5
24+	24	60
Missing	7	17.5
**Number of study visits**		
1–2	5	12.5
3–4	4	10
4+	17	42.5
Missing	14	35
**Study visit duration (minutes)**		
0–30	1	2.5
31–60	23	57.5
61–90	2	5
90+	7	17.5
Missing	7	17.5
**Study visit time of day**		
Morning	20	51.3
Lunchtime	7	17.9
Afternoon	12	30.8
Missing	1	2.5
**Study reminders**		
Yes	35	87.5
No	0	0
Missing	5	12.5
**Preferred contact method**		
Text Message	29	49.2
Email	23	39.0
Phone Call	6	10.2
Mail	1	1.7
**Advanced reminder (days)**		
1–2	12	30
3–5	3	7.5
6–10	17	42.5
10+	7	17.5
Missing	1	2.5

^a^17.5% of data were missing from study duration and duration of study visit variables, 35% of data were missing from number of study visits variable, 12.5% of data were missing from study reminders variable, and 2.5% of data were missing from advanced reminder variable.

^b^Percentages do not equal 100%, as some participants indicated multiple preferences

### Facilitators to participating in research studies

Facilitators were defined as anything that would promote or motivate pregnant or breastfeeding women to participate in and complete longitudinal research studies. As described and defined in Table 3, the following five categories of facilitators emerged: (1) aspirational benefits, (2) collateral benefits, (3) direct benefits, (4) third-party influences, and (5) location of recruitment materials.

**Table 3:** Facilitators and Barriers to Research Participation among Pregnant and Breastfeeding Women^a^

Category	Sub-themes	Description of sub-themes
Facilitators	Aspirational benefit (*n* = 25)	Personal motivations for participating in research, including contributing to science, contributing to breastfeeding research, and the potential benefit to future generations.
	Collateral benefit (n = 23)	Unintended benefits stemming from participation in the study, including access to research results, and education and community engagement.
	Direct benefit (*n* = 35)	Factors arising from research participation that provide a benefit to the participant firsthand, including childcare, lactation support, and compensation or incentives.
	Third party influences (*n* = 14)	Interpersonal factors, such as a person or group of people sharing information about the study that could influence recruitment and research participation among women who are pregnant and breastfeeding. Third party influences could be online, such as friends on Facebook and membership in specific online groups, or offline, such as physicians, friends, lactation consultants, and study team members.
	Recruitment Channel (*n* = 27)	The venue or location channel or location in which the study team should place (i.e., put, leave, post) recruitment materials for prospective participants. Recruitment channels included offline locations, such as a doctor’s office, grocery store, and playgrounds, as well as online locations, such as the Internet, Facebook, or other social media.
**Barriers**	Inconveniences (*n* = 37)	Aspects of the study that made participating more difficult, including time requirements, physical discomforts, and breastfeeding schedule.
	Protocols/study risks (*n* = 19)	Specific requirements within the study or protocol that would deter research participation and retention. These included potential risks to participants and their babies and long-term breastfeeding (in)ability.
	Biological sample collection (*n* = 9)	Factors pertaining to sample collection that deterred participation, including timing of collection, ease of collection, and sample drop off.
	Third party influences (n = 20)	Interpersonal factors that were out outside of the participant and the study that would preclude research participation, such as having a newborn (e.g., newborn with a fussy temperament), having other children, and the influence of family members.

^a^Subnodes included when n ≥ 2

#### Aspirational benefits

These were personal motivations for participation based on altruism, interest in breastfeeding, and advancing science. More than half of the participants described themselves as “passionate” about and “generally interested in breastfeeding*.”* In regard to her participation in the current study and in future studies on this topic, one woman stated, *“The overall main reason was because it has to do with breastmilk, so anything that has to do with breastmilk ever I’m always interested in” (BIS008).* Women also emphasized advancing science: As one participant noted, “*I’m a real breastfeeding advocate and I’m so interested in the microbiome because I think that’s just a brand-new science right now” (BIS011).*

#### Direct benefits

The majority of participants cited these as a reason to participate in studies, for example, childcare, lactation support, and compensation. Childcare was most frequently cited. Notably, if childcare were provided by the research team, child safety and accountability were cited as critical factors to utilizing childcare during research visits. Lactation support was described as counseling on how to breastfeed that would supplement breastfeeding education received through healthcare providers. Compensation was identified as a factor that could increase participation and retention, though participants also identified non-monetary incentives, notably, “personal touches” such as birthday cards, congratulatory cards, and thank you notes.

#### Collateral benefits

These unintended benefits that would improve research participation and retention included receiving research results, education and counseling, and community engagement. Participants would be interested in receiving research results throughout the study and were most interested in information related to personal health. Educational presentations pertaining to child-maternal health and supportive environments were cited as a collateral benefit as was education related to postpartum weight loss, infant sleeping patterns, and work-life balance, among other relevant subjects.

#### External influences

These were interpersonal factors such as a person or group of people sharing information about the study. Third party sources included online sources, such as friends on Facebook, or offline sources, such as physicians, friends, lactation consultants, and study team members. Groups on Facebook and other social media platforms were also mentioned by participants as appropriate venues for recruitment. Online groups that were specific to pregnancy and breastfeeding were particularly salient, including new parent and breastfeeding support groups*.* Third-party influences served as credible referrals who contributed to retention based on existing online or offline relationships with a prospective participant(s) or membership in a specific group.

#### Recruitment channels

These were venues or locations suggested for study teams to place (i.e., put, leave, post) recruitment materials, such as a physician office, grocery store, and playground and online options such as the Internet, Facebook, or other social media platforms. For instance, participants indicated recruitment through a medical care provider office further legitimizes the study and increases likelihood of participating: “*I would probably consider that [the study] would seem legitimate. This is at my doctor’s office and … it’s probably not bogus” (PRG002).*

### Barriers to participating in research studies

Barriers were defined as anything that could hinder or preclude participation and retention in research studies. The following four categories of barriers emerged: (1) inconveniences, (2) protocol or study risks, (3) biospecimen collection, and (4) third-party influences (Table 3).

#### Inconveniences

Participants were concerned with inconvenience to their children and themselves. One participant cited “anything that detracted” from daily activities as an inconvenience: “*The time is definitely the biggest barrier just because I had this super-long to-do list of all these things I wanted to get done before I had the baby” (BIS001).* Scheduling time for a research visit was considered particularly inconvenient for breastfeeding mothers, who would find it difficult to leave their home to travel.

#### Protocol or study risks

Most mothers were highly protective of their infants and were concerned that participating in a study could somehow hurt them. Regarding participation in a mother-infant microbiome study where the protocol requested a blood draw, one mother stated she would never allow *“anything … invasive … like you have to draw blood or something, … and I know my husband would never allow that either” (BIS011).* Other protocol requirements that could deter participation in a breastfeeding, longitudinal study was the duration of commitment to breastfeeding and breastfeeding ability.

#### Biospecimen collection

Several women discussed biospecimen collection as a potential barrier. Stool collection was considered the primary barrier to participating in microbiome studies. Pregnant women discussed physical impediments, such as constipation. Low milk production and difficulty breastfeeding were also cited as barriers. Breastfeeding women mentioned the personal value of their breast milk and considered study protocols that required a large quantity of breast milk as a barrier, especially in the case of low milk production.

#### External influences

These interpersonal factors included having multiple children along with a newborn and the influence of family members. Participants described the stress and commitment involved with supporting infants in the first months as a significant barrier. Additional family members such as other children and extended family members, particularly post-birth, were also cited as potential barriers*.*

## Discussion

Collectively, our results demonstrate that women are motivated to participate in longitudinal studies focused on breastfeeding and that barriers to participating in clinical microbiome studies are driven by inconvenience related to biospecimen collection and drop-off. Our analysis identified factors that motivate research participation with a focus on breastfeeding and perinatal education. The primary factors for non-participation focused on inconvenience related to lengthy study visits and biospecimen collection. Ideas about barriers to biospecimen collection centered on stool sampling and included inadequate instructions, sample storage, and drop-off convenience. Low milk production was also cited as a factor that would likely reduce participant retention for studies that require human milk.

An important observation from our study is the focus on breastfeeding. Specifically, participants highlighted “a passion for breastfeeding” and the idea that breastfeeding mothers could benefit was a motivation to participate in research. Notably, participants also cited elements of “conditional altruism,” that is, participation would depend on the research risks [19, 20]. Altruism is a common message strategy used for clinical recruitment [21], and our findings suggest that it may be an effective strategy for recruiting women who are breastfeeding into research studies. These findings indicate that, during early life, research participation may, in part, be driven by the emotional and practical experiences of breastfeeding [22] and by potential risks to mother and infant [23].

Although our participants considered scheduling inconvenience a barrier to participating in research, women had little concern about the duration of a study. Duration of individual study visit(s), however, was a concern. The majority of women stated that study visits should last 30–60 min. Inadequate stool collection instructions and drop-off inconvenience were considered barriers to participating in research with stool sampling. Existing data on patient preferences for stool collection indicates that informational leaflets increase patient confidence in sample collection, and specimen drop-off boxes may reduce any stigma associated with sample collection [24]. Although stool collection stigma was seldom referenced by participants in this study, drop off boxes could make participating more convenient for pregnant and breastfeeding women and should be considered. Among breastfeeding women, we also found that if, milk production was perceived to be low, that would be a barrier to participating in research studies that require human milk. Lactation support and educational materials related to biospecimen collection might improve retention of breastfeeding mothers in clinical microbiome studies.

Previous work has revealed that recruitment sources and channels influence participant enrollment and retention in clinical settings [25]. In our study, acceptable sources of materials to recruit pregnant mothers planning to breastfeed included social media platforms, physician offices, prenatal healthcare providers, and lactation consultants. These findings are consistent with previous work suggesting that women use the Internet to seek out information during pregnancy [26] and that informal relationships between mothers, both face-to-face and through social media, represent important sources of social and emotional support [27]. Thus, healthcare professionals might assist in recruiting research participants.

Our study has several strengths. First, previous qualitative work within the pregnant population has focused on understanding their perspectives on antibiotic use to develop tailored perinatal health education interventions to increase knowledge, particularly using electronic health records (EHR) to provide additional information on antibiotic use [28]. This is the first qualitative study to explore the perspectives of pregnant or breastfeeding persons on participating in longitudinal clinical studies and provides significant insight into their attitudes toward sharing their own and their infants’ non-invasive biospecimen. Secondly, the mixed-methods research design provided a more comprehensive data set concerning pregnant and breastfeeding mothers’ perspectives on participating in studies involving non-invasive biospecimen sampling. Second, our analysis reached thematic saturation suggesting our sample size was adequate for thematic analysis. This study also has some limitations. First, the participants cannot be considered representative of all pregnant and breastfeeding women, which affects the generalizability of findings. For example, the majority of participants had a professional/graduate degree, previous experience with research participation, and a low pre-pregnant body mass index (BMI). Because less educated and obese women are of great interest related to potentially vulnerable infants, a similar study of their perspectives is needed. Thirdly, due to the open-ended nature of the semi-structured interviews, missing data could potentially alter response counts.

## Conclusion

In conclusion, our results demonstrate women in this study were interested in participating in clinical studies that require non-invasive biospecimen collection, and motivations to participate center on breastfeeding and the potential to make a scientific contribution that helps others. Effectively recruiting pregnant or breastfeeding participants for and retaining them in longitudinal microbiome studies requires protocols that account for participant interests and needs and consideration for their time. With the rapid increase in microbiome studies of child-maternal health that collect biospecimens, maternal perspectives on research participation is an important topic.

## Supplementary Information

**Additional file 1.**

## Data Availability

The datasets used and/or analyzed during the current study are available from the corresponding author on reasonable request.

## Abbreviations

EHR: electronic health records
BMI: body mass index

## References

[1] Frew PM, Saint-Victor DS, Isaacs MB, et al. Recruitment and retention of pregnant women into clinical research trials: an overview of challenges, facilitators, and best practices. Clin Infect Dis 2014; 59:S400–S407. Available at: https://www.ncbi.nlm.nih.gov/pmc/articles/PMC4303058/pdf/ciu726.pdf. 10.1093/cid/ciu726 PMC4303058 25425718

[2] Yan J, Liu L, Zhu Y, Huang G, Wang PP (2014). The association between breastfeeding and childhood obesity: a meta-analysis. BMC Public Health.

[3] Tamburini S, Shen N, Wu HC, Clemente JC. The microbiome in early life: implications for health outcomes. Nat Med 2016; 22:713–22. Available at: http://www.ncbi.nlm.nih.gov/pubmed/27387886. 10.1038/nm.4142 27387886

[4] Abrahamson M, Hooker E, Ajami NJ, Petrosino JF, Orwoll ES (2017). Successful collection of stool samples for microbiome analyses from a large community-based population of elderly men. Contemp Clin Trials Commun.

[5] Flint HJ, Scott KP, Louis P, Duncan SH (2012). The role of the gut microbiota in nutrition and health. Nat Rev Gastroenterol Hepatol.

[6] Mueller NT, Bakacs E, Combellick J, Grigoryan Z, Dominguez-Bello MG (2015). The infant microbiome development : mom matters. Trends Mol Med.

[7] Dominguez-Bello MG, Costello EK, Contreras M (2010). Delivery mode shapes the acquisition and structure of the initial microbiota across multiple body habitats in newborns. Proc Natl Acad Sci.

[8] Lemas DJ, Young BE, Baker PR (2016). Alterations in human milk leptin and insulin are associated with early changes in the infant intestinal microbiome. Am J Clin Nutr.

[9] Bokulich NA, Chung J, Battaglia T, et al. Antibiotics, birth mode, and diet shape microbiome maturation during early life. Sci Transl Med 2016; 8:343ra82. Available at: http://www.ncbi.nlm.nih.gov/pubmed/27306664. 10.1126/scitranslmed.aad7121 PMC5308924 27306664

[10] Azad MB, Konya T, Maughan H, et al. Gut microbiota of healthy Canadian infants: profiles by mode of delivery and infant diet at 4 months. Can Med Assoc J 2013; 185:385–394. Available at: http://www.ncbi.nlm.nih.gov/pubmed/23401405. 10.1503/cmaj.121189 PMC3602254 23401405

[11] Aagaard K, Ma J, Antony KM, Ganu R, Petrosino J, Versalovic J. The placenta harbors a unique microbiome. Sci Transl Med **2014**; 6:237ra65. 10.1126/scitranslmed.3008599 PMC4929217 24848255

[12] Koren O, Goodrich JK, Cullender TC (2012). Host remodeling of the gut microbiome and metabolic changes during pregnancy. Cell.

[13] Goodrich JK, Di Rienzi SC, Poole AC, et al. Conducting a Microbiome Study. Cell 2014; 158:250–262. Available at: http://www.ncbi.nlm.nih.gov/pubmed/25036628. 10.1016/j.cell.2014.06.037 PMC5074386 25036628

[14] Lemas DJ, Yee S, Cacho N (2016). Exploring the contribution of maternal antibiotics and breastfeeding to development of the infant microbiome and pediatric obesity. Semin Fetal Neonatal Med.

[15] Lecky DM, Nakiboneka-Ssenabulya D, Nichols T, et al. Informing future research for carriage of multiresistant gram-negative bacteria: problems with recruiting to an English stool sample community prevalence study. BMJ Open. 2017;7. 10.1136/bmjopen-2017-017947 PMC5778273 29229656

[16] Smith JA (1995). Chapter 2: semi-structured interviewing and qualitative analysis.

[17] Dougherty D. Grounded theory research methods. In: The Blackwell companion to organizations. Oxford, UK: Blackwell Publishing Ltd, 2017: 849–866. Available at: http://doi.wiley.com/10.1002/9781405164061.ch37.

[18] Bazeley P (2013). Qualitative data analysis: practical strategies.

[19] Kenyon S, Dixon-Woods M, Jackson CJ, Windridge K, Pitchforth E (2006). Participating in a trial in a critical situation: a qualitative study in pregnancy. Qual Saf Heal Care.

[20] McCann SK, Campbell MK, Entwistle VA. Reasons for participating in randomised controlled trials: conditional altruism and considerations for self. Trials 2010; 11:31. Available at: http://www.ncbi.nlm.nih.gov/pubmed/20307273. 10.1186/1745-6215-11-31 PMC2848220 20307273

[21] Flood-Grady E, Paige SR, Karimipour N, Harris PA, Cottler LB, Krieger JL. A content analysis of Clinical and Translational Science Award (CTSA) strategies for communicating about clinical research participation online. J Clin Transl Sci 2017; 1:340–351. Available at: http://www.ncbi.nlm.nih.gov/pubmed/29707256. 10.1017/cts.2018.2 PMC5915806 29707256

[22] Fallon V, Komninou S, Bennett KM, Halford JCG, Harrold JA. The emotional and practical experiences of formula-feeding mothers. Matern Child Nutr. 2017;13. 10.1111/mcn.12392 PMC6866173 27862970

[23] Smyth RMD, Jacoby A, Elbourne D (2012). Deciding to join a perinatal randomised controlled trial: experiences and views of pregnant women enroled in the magpie trial. Midwifery.

[24] Lecky DM, Hawking MKD, McNulty CAM. Patients’ perspectives on providing a stool sample to their GP: A qualitative study. Br J Gen Pract 2014; 64:e684–e693. Available at: http://www.ncbi.nlm.nih.gov/pubmed/25348992. 10.3399/bjgp14X682261 PMC4220220 25348992

[25] Flood-Grady E, Clark VC, Bauer A (2017). Evaluating the efficacy of a registry linked to a consent to re-contact program and communication strategies for recruiting and enrolling participants into clinical trials. Contemp Clin Trials Commun.

[26] Sayakhot P, Carolan-Olah M. Internet use by pregnant women seeking pregnancy-related information: a systematic review. BMC Pregnancy Childbirth. 2016;16. 10.1186/s12884-016-0856-5 PMC4810511 27021727

[27] Zimmerman TS, Aberle J, Krafchick J, Harvey A (2008). Deconstructing the ‘mommy wars’: the battle over the best mom. J Fem Fam Ther.

[28] Chen LY, Flood-Grady E, Hentschel A, et al. A qualitative study of pregnant Women’s perspectives on antibiotic use for mom and child: implications for developing tailored health education interventions. Antibiotics 2020; 9:704. Available at: https://www.mdpi.com/2079-6382/9/10/704. 10.3390/antibiotics9100704 PMC7602878 33076539

