# Supplementary material for: Perspectives of pregnant and breastfeeding women on longitudinal clinical studies that require non-invasive biospecimen collection – a qualitative study
Source: BMC Pregnancy Childbirth. 2021 Jan 20;21:67. doi: 10.1186/s12884-021-03541-x (PMC7816422; doi:10.1186/s12884-021-03541-x)
Supplement: Supplementary file 1 — Additional file 1. [file 12884_2021_3541_MOESM1_ESM.docx]

**SUPPLEMENTARY INFORMATION**


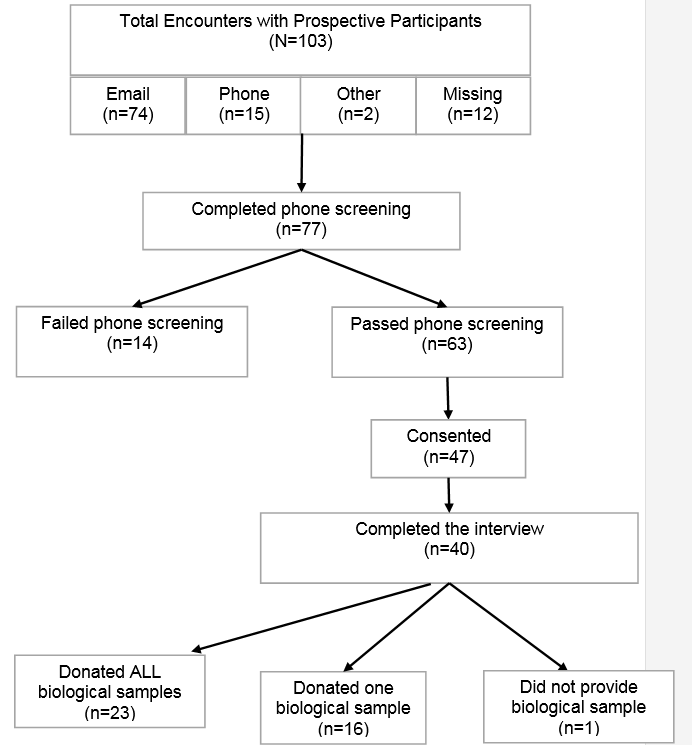


**Supplementary Figure 1.** Overview of recruitment strategies and participation for study population. Encounters refer to contacting prospective participants about the study.
